# Can Stabilization and Inhibition of Aquaporins Contribute to Future Development of Biomimetic Membranes?

**DOI:** 10.3390/membranes5030352

**Published:** 2015-08-10

**Authors:** Janet To, Jaume Torres

**Affiliations:** School of Biological Sciences, Nanyang Technological University, 60 Nanyang Drive, Singapore 637551, Singapore; E-Mail: YYTO1@e.ntu.edu.sg

**Keywords:** aquaporins, biomimetic membranes, stable mutants, aquaporin inhibitors, high-throughput assays

## Abstract

In recent years, the use of biomimetic membranes that incorporate membrane proteins, *i.e.*, biomimetic-hybrid membranes, has increased almost exponentially. Key membrane proteins in these systems have been aquaporins, which selectively permeabilize cellular membranes to water. Aquaporins may be incorporated into synthetic lipid bilayers or to more stable structures made of block copolymers or solid-state nanopores. However, translocation of aquaporins to these alien environments has adverse consequences in terms of performance and stability. Aquaporins incorporated in biomimetic membranes for use in water purification and desalination should also withstand the harsh environment that may prevail in these conditions, such as high pressure, and presence of salt or other chemicals. In this respect, modified aquaporins that can be adapted to these new environments should be developed. Another challenge is that biomimetic membranes that incorporate high densities of aquaporin should be defect-free, and this can only be efficiently ascertained with the availability of completely inactive mutants that behave otherwise like the wild type aquaporin, or with effective non-toxic water channel inhibitors that are so far inexistent. In this review, we describe approaches that can potentially be used to overcome these challenges.

## 1. General Features of Aquaporins

**Figure 1:**
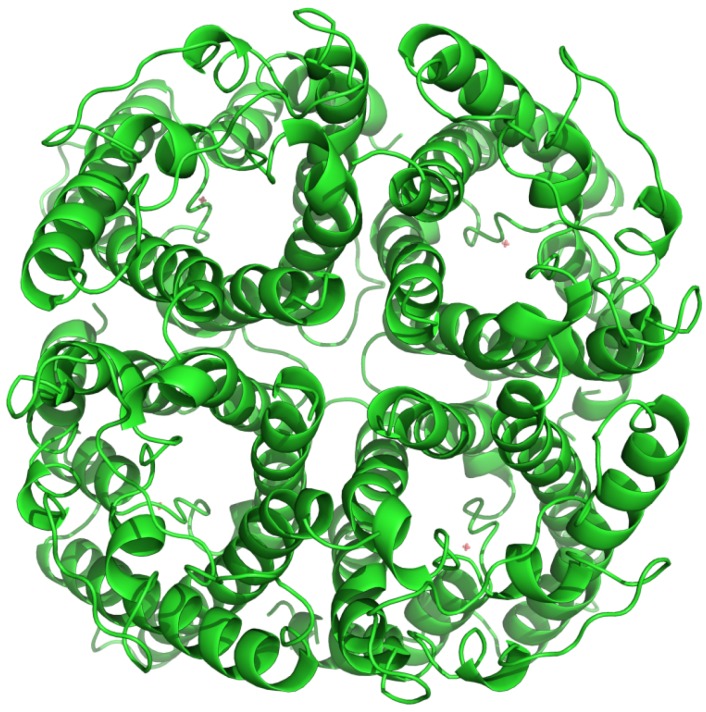
General structure of aquaporins, with a tetrameric arrangement of water channels. This structure is for human AQP1.

Aquaporins (AQPs) are integral membrane proteins that transport water through cellular membranes [1,2,3]. In humans, there are 13 different AQPs [4], from AQP0 to AQP12. The last 15 years have revealed important physiological roles for AQPs (reviewed in [5]), not surprisingly related to their water channel activity, e.g., in the urinary concentrating mechanism, glandular fluid secretion, or brain swelling. The architecture of AQPs is remarkably conserved from bacteria to humans [6,7,8,9,10,11,12]: the functional form is a homo-tetramer, where each AQP monomer has six transmembrane (TM) α-helical domains and functions independently as a water channel, e.g., human AQP1 [13] (Figure 1). Each monomer can transport as many as 3 billion water molecules per second, while rejecting all other solutes, including protons [14]. Such high permeabilities and selectivities depend on geometric and physico-chemical factors that are now beginning to be understood, as recently shown for a yeast aquaporin [15].

The availability of functional AQP proteins of high purity has helped tremendously in the high-resolution X-ray structural determination of important AQPs: *Escherichia coli* orthodox AqpZ [16] and glycerol facilitator GlpF [17], archaeal AqpM [11], mammalian AQP0 [18], AQP1 [7], AQP2 [19], AQP4 [20] and AQP5 [9], as well as spinach SoPIP2;1 [21]. The use of solid-state NMR may also be useful for structural studies of AQPs in the near future, as suggested by high resolution spectra obtained for hAQP1 [22]. Recombinant AQP proteins are commonly expressed from several host organisms, including the bacterial *E. coli* [23], mammalian insect *Spodoptera frugiperda* Sf9 [24], yeast *Pichia pastoris* [25], and *Saccharomyces cerevisiae* [26]. Typically, a target AQP encoded by a host expression vector will be expressed in fusion with an affinity tag, commonly a polyhistidine (6His) tag. Once this recombinant AQP is produced from host cells, host lysis is closely followed by reconstitution of the recombinant AQP from host cell membranes into stabilizing biomimetic environments, *i.e.*, detergent micelles. This will provide a suitable environment for downstream affinity purification of the recombinant AQP using nickel-nitrilotriacetic acid (Ni-NTA) chromatography.

The addition of recombinant fusion partners such as the maltose binding protein (MBP), and the optimization of codon usage has been shown to boost production levels of AqpZ to 200 mg/L of *E. coli* culture [27]. Similarly, protein yields of human AQPs expressed from *P. pastoris* can also be improved by codon optimization and clone selection [28]. In addition, high production of AQP proteins is also possible using cell-free (CF) expression system, a novel approach which mimics the natural cell cytoplasmic environment for protein synthesis. This holds advantages over traditional *in vivo* membrane protein expression in living cells, such as elimination of toxicity to host cell physiology due to membrane incorporation of recombinant proteins, and overloading of essential cellular protein-targeting machineries in order to overexpress a foreign protein [29]. CF extracts can be obtained from *E. coli* cells, wheat germs, rabbit reticulocytes, insect cells, and more recently, Chinese hamster ovary (CHO) cells [30]. In the CF system, newly synthesized AQP proteins can be incorporated directly into artificial hydrophobic environments, like detergent micelles [31] or synthetic liposomes [32]. By introducing isotopically-labelled or fluorescence-enhanced amino acids into the reaction mixture, efficient labelling of the target AQP is also possible.

## 2. Aquaporins in Biomimetic Membranes

The exquisite permeability and selectivity of aquaporins to water made them a unique component in the development of water filtration devices. Since a landmark paper in 2007 [33], development of desalination membranes based on AQPs (*E. coli* aquaporin Z, AqpZ) has attracted much attention globally. Research in this field has grown over the last few years, e.g., [34,35,36,37,38]. Recent reviews are available for aquaporin-based biomimetic membranes [39,40] and for biomimetic membranes in general [41].

For example, significant improvements have been achieved using AqpZ-based proteoliposomes in a cross-linked polyamide matrix by interfacial polymerization [38], but water permeability is still modest compared to their expected values. One possible cause is the negative impact of the chemicals used for interfacial polymerization on the activity of AQPs in lipid membranes (proteoliposomes). In addition, the loading amount of proteoliposome in the selective layer is relatively low. In addition, membrane defect minimization depends on chemical modification of the membrane, which may, in turn, have a negative impact on AQP function.

Amphiphilic block copolymers (BCPs) have also been used as substitutes of lipids. These assemble into bilayer-like structures [42] that have superior properties when compared to lipids, e.g., higher mechanical and chemical stability and low water and gas permeability. In addition, the geometric and chemical characteristics of these membranes can be customized. The use of different BCPs is an alternative strategy, but, so far, tests are limited to a single polymer type, polydimethylsiloxane (PDMS) hydrophobic block. Alternatively, functional AqpZ mutants could be screened for compatibility and insertion efficiency with a given polymer type. These mutants may also lead to higher expression levels, which is another fundamental challenge for large-scale applications. While high yields have been reported [43] in *E. coli*, these were obtained from a fusion form, which may require further purification steps. 

Additionally, although AQPs other than AqpZ have been incorporated into BCP membranes with success [44], the requirements for compatibility between membrane protein and polymer are not well understood, therefore a rational basis for polymer selection does not exist.

A successful biomimetic membrane would require high levels of protein packing in membranes, compared to the low levels achieved so far for aquaporins in BCP membranes. For AqpZ in poly-(2-methyloxazoline)-block-poly-(dimethylsiloxane)-block-poly-(2-methyloxazoline) (PMOXA-PDMS-PMOXA) triblock copolymer membranes, a molar polymer to protein ratio of 50–100 was obtained, beyond which permeability decreased [33]. Optimization of these parameters can be achieved with the availability of robust and functional AQP mutants, which can withstand a wider range of chemical modifications, while at the same time increase shelf life and potentially increase insertion efficiency and packing. Another exciting achievement would be to obtain AQP mutants that can withstand cross-linking with lipids in order to stabilize membranes, without being denatured [45] while still retaining permeability and selectivity.

These AQP mutants should have increased stability while obviously retaining their function. Fortunately, it is not difficult to obtain stabilizing mutations in a membrane protein, while retaining function [46,47,48]. However, at present, it is impractical to engineer these mutants based only on theoretical principles and available detailed structural data [15]. Generic assays to select thermostable mutants of proteins have been recently reported [49], but these mutants have to be tested for function later, in a high-throughput manner. This is especially challenging for aquaporins, which do not have easily testable enzymatic or binding activities. In the following sections, we will provide an overview on the current assays used to study aquaporin function with special emphasis on a recently developed assay for the identification of functional AQP mutants and how these mutants can be further characterized for protein stability.

## 3. Functional Assays for Aquaporins

Currently known functional assays for AQPs described in the literature are summarized in Table 1, along with their ease of use and high-throughput capabilities.

### 3.1. Stopped-Flow Water Permeability Assay

The kinetics of cell volume changes in response to rapidly imposed osmotic gradients can be monitored to quantify water permeability through membranes containing AQPs. This can be performed using suspended cells (e.g., erythrocytes which express native AQP1), plasma membrane vesicles obtained from AQP-expressing cells, or artificial liposomes (of a defined size) reconstituted with purified AQP proteins (proteoliposomes) [50]. Typically, the rate of cell or vesicle swelling or shrinking due to rapid hypotonic or hypertonic osmotic challenges can be studied by following changes in light scattering intensity. Alternatively, vesicles can be pre-loaded with fluorophores such as carboxyfluorescein, where changes in intravesicular fluorescence caused by the self-quenching of entrapped carboxyfluorescein as a result of water efflux can be measured [51]. Water flow measurements require specialized instrumentation, *i.e.*, the stopped-flow spectrometer [52]. For instance, the rapid mixing of a cell suspension with a hyperosmolar solution (containing a nonpermeant osmolyte such as sucrose) will generate an osmotic gradient to drive water efflux, and the rate of cell shrinkage is measured based on the change in intensity of 90° scattered light (λ = 500 nm), as a function of time, allowing for calculation of osmotic water permeability (*P*_f_) [53].

**Table 1:** Currently known aquaporins (AQP) water transport functional assays.

Assay	System	Readout	Throughput	Characteristics	References
Stopped-flow water permeability assay	Suspended AQP-proteoliposomes, vesicles or cells (e.g., erythrocytes)	Light scattering or fluorescence changes	Low; about 10 samples per hour	Requires specialized instrumentation *i.e.*, stopped-flow spectrometer	[ 52,53]
Transepithelial assay	Cell monolayers cultured on porous support	Dilution of indicator dye	Low; 12 wells per plate	Virtually free from artifacts Laborious to perform	[ 54]
Fluorescence-based assay	Cell monolayers cultured on solid support	Cytoplasmic fluorescence changes	Medium; 96-well plates	Potential artifacts related to interaction of compound with reporter fluorescence Easy to perform	[ 55–57]
Oocyte swelling assay	Oocytes from *Xenopus laevis*	Oocyte imaging	Low; about 2–5 samples per day	Prone to artifacts Technically challenging	[ 3]
Erythrocyte lysis assay	Erythrocytes	Cell lysis	High; 96-well plates	Only applicable for AQP1	[ 58,59]
Yeast freeze-thaw assay	Yeast cells	Cell viability	High; 96-well plates	Generic Easy to perform	[ 60]

### 3.2. Transepithelial Assay

Mammalian transepithelial assays measure water flux across a tight cell layer cultured on a porous support, with a typical transepithelial resistance of 1–2 kΩ·cm^2^ [54]. The cells under study can either be expressing endogenous AQPs, or to be stably transfected with plasmids encoding AQPs. In this assay, transepithelial water permeability driven by an osmotic gradient using membrane-impermeable solutes such as mannitol or sucrose is measured using a dye dilution method. Measurements of the rate of fluorescence changes of an indicator dye in the apical fluid volume enable a quantitative readout of water flux across the epithelial cell layer in order to compute the transepithelial osmotic water permeability coefficient (*P_f_*). Although this method is sensitive and virtually free from artifacts, it requires expertise in the use of cells and is therefore recommended to be used as a secondary assay to verify hits obtained from primary high-throughput screening campaigns.

### 3.3. Fluorescence-Based Assays

Cell volume changes can be studied by monitoring changes in the intensity of fluorescence reporter dyes in fluorescently-labelled cells. In the calcein method, cells cultured on a solid support, such as a 96-multiwell fluorescence plate format, are loaded with membrane-permeable calcein-acetomethoxy derivate (calcein-AM), which gets trapped intracellularly due to cleavage by cellular esterases. The screening strategy is based on the kinetics of concentration-dependent self-quenching of calcein fluorescence in response to cell shrinkage. Upon rapid exposure to a hypertonic stimulus, water leaves the cells through AQPs, leading to changes in fluorescence which is directly proportional to changes in cell water volume [55]. Since the assay can be performed multiwell plate format, it has the potential for lab automation. A similar fluorescence-based method uses genetically-encoded, cytoplasmically expressed fluorescent proteins, *i.e.*, a mutant yellow fluorescence protein, YFP-H148Q/V163S, whose fluorescence is quenched by chloride [56]. Using this fluorescence chloride sensor, osmotic water permeability can be measured from the kinetics of decreasing cytoplasmic chloride concentrations resulted from an osmotically induced cell swelling. In order for the kinetics of cell volume changes to be measured accurately, the osmotic response of the cells has to be sufficiently slow (~few seconds), while the time taken for solution mixing should be short relative to the time course of osmotic equilibration.

### 3.4. Oocyte Swelling Assay

The *Xenopus laevis* oocyte swelling assay is the original functional assay used to demonstrate that AQP1 functions as a water channel [3]. In this system, defolliculated oocytes are microinjected with *in vitro*-transcribed AQP1 capped RNA (cRNA) to induce expression of AQP1 at the oocyte membrane. After ~72 h incubation, the oocyte will be transferred from an isotonic medium to a hypotonic medium, and oocyte volume changes will be followed with video microscopy using sequential oocyte images photoed at 15 s intervals for a total of 5 min or until just before the rupture of oocyte membrane. The osmotic water permeability *P*_f_ can be determined from the time-course changes in relative oocyte volume [3].

### 3.5. Erythrocyte Lysis Assay

A high-throughput method, based on the lysis of erythrocytes, has previously identified inhibitors of the urea transporter B (UT-B) of nanomolar potency [58], and can be improvised to screen for AQP inhibitors [59]. Human erythrocytes, which express endogenous AQP1 and urea transporter UT-B, are first pre-loaded with the urea analog acetamide, which is transported by UT-B and equilibrates across the erythrocyte membrane but at a slightly slower rate compared to water. The dilution of these acetamide-loaded erythrocytes into an acetamide-free solution results in a rapid AQP-dependent influx of water, cell swelling and subsequent lysis. If the AQP is not functional, or in the presence of an AQP1 inhibitor, the rate of water influx is reduced, thereby allowing the dissipation of the osmotic gradient by efflux of acetamide. As a result, cell lysis is prevented. Extent of cell lysis can be quantified by single time point readout of near-infrared light absorbance at 710 nm wavelength.

### 3.6. Yeast Freeze-Thaw Assay

This assay is based on the protective effect of AQPs on yeast in response to rapid freezing challenge. This effect was observed in earlier studies of the baker’s yeast *Saccharomyces cerevisiae,* where a correlation was shown to exist between freeze-resistance and expression of yeast aquaporins AQY1 and AQY2; deletion of AQP-encoding genes made yeast more sensitive to freezing, whereas overexpression of AQPs (yeast AQY1/AQY2 or human hAQP1) improved their freeze-thaw resistance [61,62]. Apparently, AQPs permit a rapid efflux of water through the yeast membrane during freezing, reducing intracellular ice crystal formation and thereby rescuing the cell from damage upon thawing [61,62]. This has been the basis to develop a generic high-throughput assay [60] that is in principle applicable to AQPs of any organism as long as they transport water.

**Figure 2:**
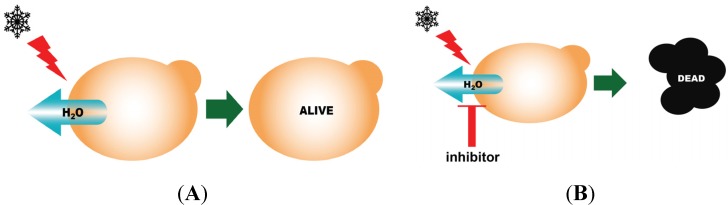
Schematic representation of the yeast-based freeze-thaw assay. If the yeast expresses an active AQP, they will survive a freeze-thaw challenge (**A**); If an inactive AQP is expressed, or if an active AQP expressed is exposed to an AQP inhibitor, the protective effect is lost and the yeast will die (**B**).

**Figure 3:**
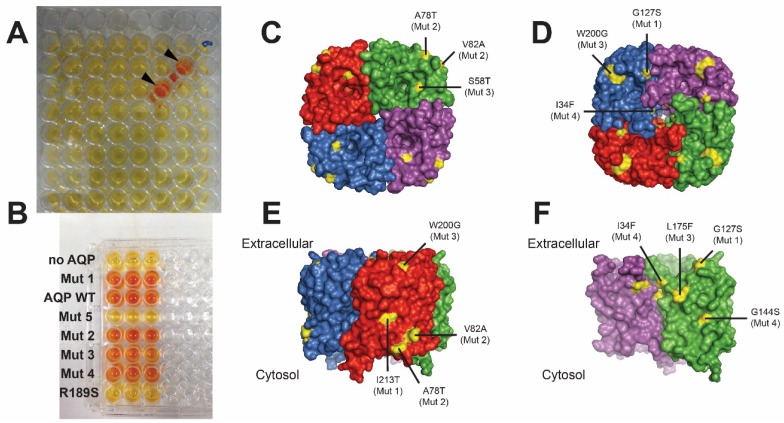
Selection of functional AqpZ mutants. (**A**) Screening of a 96-well plate with yeast expressing AqpZ mutants, showing two wells with water-permeable AqpZ (arrows). Conversion of added reagents to orange colour indicates presence of viable cells; (**B**) Re-screening comparing yeast without AQP or expressing wild type AqpZ (WT), an inactive mutant (R189S) and several inactive/active mutants found; (C-F) Nine functional mutations (yellow), out of 160 mutants carrying 1–5 mutations each, shown on the structure of the AqpZ tetramer: view from (**C**) cytoplasmic and (**D**) extracellular side; (**E,F**) side view (**E**) and after removing two monomers (**F**). Results adapted from [60].

In this assay, yeast cells lacking native AQPs but overexpressing an AQP of choice are exposed to freeze-thawing. Only cells expressing functional water-permeable AQPs are rescued from the challenge. Yeast expressing inactive AQPs, or yeast expressing active AQPs but exposed to AQP inhibitors, are not protected which results in cell death (see schematic representation of this method in Figure 2). Identification of functional AQP mutants can be achieved by generating first a library of random AQP mutants, which are then tested for viability after a freeze-thawing challenge (Figure 3) [60].

**Figure 4:**
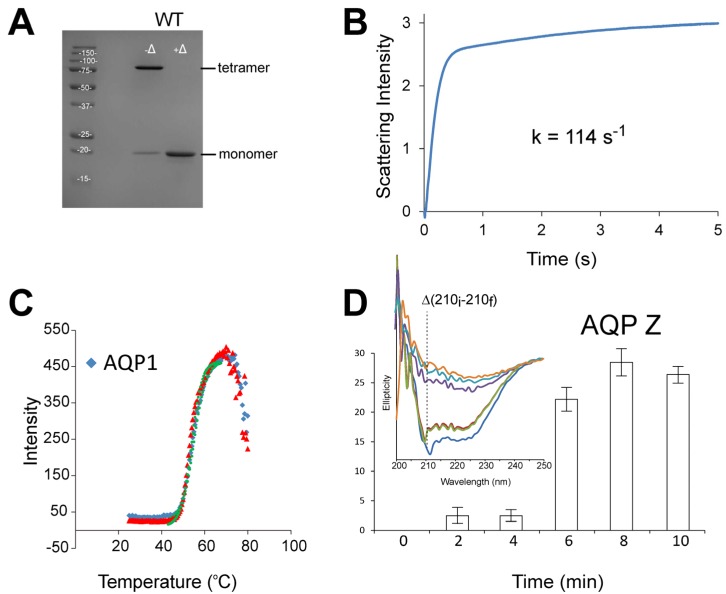
Aquaporin thermostability and functional assays. (**A**) In SDS, AqpZ is tetrameric at room temperature −Δ but monomeric at higher temperature +Δ; (**B**) Stopped-flow water permeability assay, where light scattering is continuously recorded after mixing AqpZ proteoliposomes with a high sucrose solution; (**C**) TSAdata of hAQP1 for two sample repeats (blue and red) obtained using StarGazer-384. Data collected can be analysed using the Bioactive software (Harbinger Biotech), where the measured scattering intensities are plotted as a function of temperature and fitted to the Boltzmann equation by non-linear regression (green line). The temperature of aggregation (T_agg_) is defined as the point of inflection (dotted lines)Shift of T_agg_ to a higher temperature indicates an increase in protein thermostability; (**D**) Unfolding of AqpZ, measured as decrease in ellipticity, when the protein is heated at constant temperature, e.g., 78 °C. In this case, the loss of α-helix content is measured at 210 nm, and expressed as the difference between the initial ellipticity at time zero (210_i_) and the ellipticity after a given time (210_f_).

One further advantage of this method is that these mutants may then be tested for thermostability while still in the yeast, avoiding lengthy expression and purification steps. This is possible because native AqpZ is tetrameric in detergent sodium dodecyl sulfate (SDS) (unlike hAQP1, which is monomeric) but forms monomers when heated (Figure 4A). In contrast, native hAQP1 forms monomers in SDS at room temperature, hence hAQP1 mutants with higher stability may retain their tetrameric form at room temperature. Therefore, a Western blot analysis on yeast cell lysates can detect increased thermal stability in AqpZ and resistance to SDS denaturation in AQP1, bypassing purification steps. Following this workflow, only those promising mutants would be then analyzed in detail using downstream biophysical assays, e.g., analytical ultracentrifugation sedimentation velocity (SV) to determine monomer-tetramer association constants.

## 4. Stability of Aquaporins

Assessment of protein thermal and chemical stability provides an indication of how the protein will behave under adverse conditions. The thermal stability of AQPs can be studied using the thermal shift assay (TSA) [60,63], which is based on differential static light scattering (DSLS). The latter can be measured using the StarGazer-384 (Harbinger Biotechnology, Toronto, Canada), without interference from detergents present in the system [64]. This assay estimates protein stability by monitoring protein aggregation and increase in scattering upon step-wise heating. Typically, samples are heated from 25 to 80 °C, at a rate of 1 °C per minute (Figure 4C). In this case, AQP mutants with enhanced thermostability would produce a higher protein’s aggregation temperature (T_agg_) compared to the wild type. TSA can be easily scaled up to a 384-well format to simultaneously assess the thermostability of a large library of AQP mutants.

Native AqpZ shows a much higher T_agg_ than hAQP1 (>80 °C *vs.* ~55 °C), therefore the detection of thermal shift using this method is more difficult for AqpZ due to evaporation problems. Nevertheless, CD can be used instead to show that after brief exposure to a high temperature (78 °C) AqpZ starts unfolding in a few minutes (Figure 4D), allowing quantification of its stability. AQP thermal unfolding in different detergents and lipid environments has been previously followed using CD thermal denaturation assay, e.g., spinach AQP SoPIP2;1 [65], where it was found that the incorporation of the AQP into *E. coli* lipid membranes increased the melting temperature of the protein.

In addition, chemical stability of AQP mutants can be tested by exposure to a variety of environmental conditions, e.g., extreme pH or oxidants, or crosslinking with lipid-like molecules following established protocols [45].

## 5. Aquaporin Inhibitors

Selective inhibitors of AQPs serve as invaluable negative control tools to quantify water permeability through AQP-based biomimetic membranes, aside from the obvious benefits for drug discovery since AQPs are the new players in cancer biology [66], e.g., human AQP1 [67,68,69]. However, despite numerous reports describing the discovery of agents that can modulate water flux through AQPs, they are not suitable for drug discovery efforts, mainly due to their toxic side effects and/or lack of selectivity and potency [70,71,72,73,74,75,76,77,78,79,80,81,82,83,84]. A discussion of various proposed AQP inhibitors and their molecular structures can be found in Figure 5 of reference [59]. Mercurial compounds, although notorious for their toxic properties, are the first blockers of water permeability through AQPs to be described in the literature. Inhibition of AQP1 by sulfhydryl-reactive mercurials such as mercury (II) chloride involves a covalent interaction of the Hg^2+^ with the Cys189 residue at the extracellular side, leading to a steric block of the water channel. Site-directed mutagenesis studies demonstrated that mutation in the Cys189 of AQP1 prevent inhibition by mercury in a *Xenopus* oocyte swelling assay [72], while other AQPs (e.g., AQP4) which lack the cysteine residue at that position are shown to be resistant to Hg^2+^ inhibition. Similarly, a mutant of AqpZ (T183C) engineered based on the known mercury-sensitive site of AQP1, rendered the channel sensitive to inhibition by Hg^2+^ [12]. Other heavy metals such as silver (as silver nitrate or silver sulfadiazine) [74], gold(III) (in compounds Auphen, Auterpy, Aubipy, AubipyMe and AubipyNH_2_) [85] and zinc (as zinc chloride) [79] are also able to inhibit several AQP isoforms. Additionally, other agents such as derivatives of loop diuretic bumetanide [80], TGN-020 (2-(nicotinamide)-1,3,4-thiadiazole) [86] and the general anesthetic propofol [87] have also been reported to inhibit AQPs.

Quaternary ammonium salts such as tetraethyl ammonium chloride (TEA), a known pore-occluding blocker of voltage-gated potassium ion channels [88], was reported to inhibit the water permeability through AQP1 [76,77,78]. Based on the resistance of an AQP1 mutant that carries a mutation at the Tyr186 site, the binding site for TEA+ ions was proposed to be through interactions with the Loop E pore region [76]. It has been described that acetazolamide (AZA), a pan-inhibitor of carbonic anhydrases, inhibits AQP1 in a *Xenopus* oocyte swelling assay [82], and also in human embryonic kidney (HEK293) cells transfected with AQP1 in a green fluorescence protein (GFP) fluorescence intensity assay [89]. In the latter study, the direct binding of AZA to AQP1 was also detected by biomolecular interaction analysis based on Surface Plasmon Resonance, with an equilibrium constant (K_D_) of ~1 × 10^−4^ M. However, despite these reported efficacies in blockage of water transport through AQP1-expressing *Xenopus* oocytes, the Verkman lab has demonstrated that neither TEA nor AZA could inhibit AQP1 in human erythrocyte stopped-flow and transepithelial water permeability measurements, even when tested at 10 mM (for TEA) or 2 mM (for AZA due to solubility limit) concentrations [70]. Later on, an improved *Xenopus* oocyte system also did not observe any effects of TEA or AZA on the water permeability of AQP1 [90]. It was suggested that the false positive results obtained previously could be due to secondary effects related to changes in membrane conductance and intracellular osmolarity throughout the time-course of oocyte swelling measurements, which are independent of direct interactions between AQP1 and the putative inhibitors.

A medium-throughput primary screening assay based on calcein self-quenching used mammalian cells expressing AQP1 and AQP4 to test a library of 3,575 compounds from the National Cancer Institute, including 418 FDA-approved drugs, for effects on water transport [57]. After a secondary evaluation of initial hits using stopped-flow water permeability assay on plasma membrane vesicles obtained from AQP4-transfected cells and rat erythrocytes expressing endogenous AQP1, the campaign yielded four hit compounds, NSC164914, NSC670229, NSC168597 and NSC301460, with EC_50_ values of ~27–49 µM when tested on rat erythrocytes.

More recently, the screening of a library of ~10,000 drug-like compounds against the human AQP1 using the yeast freeze-thaw assay has been completed, yielding putative inhibitors which can block water permeability through human erythrocytes [60]. The effectiveness of these hits on purified hAQP1 was also assessed using several biophysical methods to characterize binding, stabilization and inhibition of the protein. Two novel hAQP1 inhibitors, NSC670226 and NSC657298, were identified by this screening effort.

In a virtual screening effort by molecular docking of lead-like subset (1,000,000 compounds) against the extracellular part of hAQP1, 14 compounds from the top 50 list were experimentally in an hAQP1-cRNA injected *Xenopus* oocyte swelling assay [91]. In the same study, the inhibition by AZA produced an IC_50_ of 5.5 µM, although it has been previously. Three hit compounds showed reduction in osmotic swelling, with IC_50_ values of ~8–17 µM. Through molecular dynamics simulation studies, the Lys36 residue was observed to interact with these three compounds in some way. This was supported by frog oocyte swelling assays on a hAQP1-K36A mutant, which could not be inhibited by these hit compounds, but can still be inhibited by acetazolamide. Unfortunately, none of these three compounds were effective in inhibiting water transport when tested in a human erythrocyte stopped-flow water permeability assay. The authors suggested that such discrepancy could be due to lower protein expression levels in frog oocytes compared to that in human erythrocytes.

## 6. Conclusions

Many challenges must be overcome before the use of aquaporin-based biomimetic membranes can be considered mainstream. For example, systematic research is required to understand the interactions and compatibility between aquaporin and matrix materials. There is also a lack of understanding of the factors affecting long-term stability of biomimetic hybrid assemblies. In turn, these will determine scalability and production costs [41]. Despite these challenges, this promising area of research presents major opportunities for paradigm shift technologies in areas most critical to human health, quality of life and the environment.

Apart from constructing biomimetic membranes based on traditionally used *E. coli* AqpZ, other aquaporin isoforms may also be tested for higher permeability and/or better insertion efficiency in BCPs. However, given that aquaporins have adapted specifically to their natural environments, a more logical approach would be to create mutant forms of aquaporins adapted to these alien biomimetic environments. At the same time, these mutants could be selected for higher expression compared to the wild type. Robust mutants are expected to improve on the various aspects that are critical for these biomimetic membranes to be taken from its present small-scale format to much desired large-scale industrial applications. They are: extended shelf- and operational- life, resistance to chemicals, compatibility with synthetic polymers, as well as packing and production yields.

Protein thermal stability correlates with desirable features such as increased shelf life, robustness and enhanced crystallizability. Two main practical applications are proposed for these mutants: robust components of biomimetic membranes used for water purification and formation of protein crystals of superior X-ray diffraction properties. For the first application, the orthodox aquaporin from *E. coli* (AqpZ) is the current workhorse for biomimetic membrane development. Availability of robust mutants will accelerate the transition from small-scale format to large-scale industrial applications, via extended shelf- and operational- life, and possibly insertion efficiency and resistance to harsh chemical treatments. 

Selective inhibitors, including naturally occurring toxins and organic molecules, have been identified and characterized for classical membrane proteins such as ion channels, membrane receptors and transporters. Unfortunately, specific efficient inhibitors are lacking. The progress in this field has been unexpectedly slow, owing to the poor druggability of aquaporins and the lack of gold-standard assays, in contrast to the electrophysiological assays that are available for ion channels. Nevertheless, continual advances in aquaporin-based research may eventually deliver a breakthrough in this area. 
